# Analysis of multiple bone responses to graded strains above functional levels, and to disuse, in mice *in vivo* show that the human Lrp5 G171V High Bone Mass mutation increases the osteogenic response to loading but that lack of Lrp5 activity reduces it

**DOI:** 10.1016/j.bone.2011.03.683

**Published:** 2011-08

**Authors:** Leanne K. Saxon, Brendan F. Jackson, Toshihiro Sugiyama, Lance E. Lanyon, Joanna S. Price

**Affiliations:** a Department of Veterinary Basic Sciences, The Royal Veterinary College, University of London, London NW1 0TU, UK; b Department of Veterinary Clinical Sciences, The Royal Veterinary College, University of London, UK; c School of Veterinary Sciences, University of Bristol, Bristol BS40 5DU, UK

**Keywords:** *Lrp5*, Bone, Mechanical loading, Disuse, MicroCT

## Abstract

**Introduction:**

To investigate the role of the low-density lipoprotein receptor-related protein 5 (*Lrp5*) in bones' responses to loading, we analysed changes in multiple measures of bone architecture in tibias subjected to loading or disuse in male and female mice with the *Lrp5* loss of function mutation *(Lrp5*^*−/−*^) or heterozygous for the *Lrp5* G171V High Bone Mass (HBM) mutation (*Lrp5^HBM+^*).

**Materials and methods:**

The right tibias of these 17 week old male and female mice and their Wild Type (WT) littermates were subjected to short periods of loading three days a week for two weeks. Each tibia was loaded for 40 cycles, to produce peak strains at the midshaft within the low, medium or high physiological range (~ 1500, 2400 and 3000 microstrain, respectively). In similar groups of mice the right sciatic nerve was severed causing disuse of the right tibia for 3 weeks. Data from microCT of loaded, neurectomised and contra-lateral control tibias were analysed to quantify changes in the cortical and cancellous regions of the bone in the absence of functional strains and in response to graded strains in addition to those derived from function.

**Results and conclusion:**

Male *WT*^*+/+*^ controls showed significant strain:response curves for cortical area and trabecular thickness, but *Lrp5*^*−/−*^ mice showed no detectable strain:response in those same outcomes. Female mice of either *WT*^*+/+*^ or *Lrp5*^*−/−*^ genotype did not show significant strain:response curves for cortical or trabecular parameters, the one exception being Tb.Th in *Lrp5*^*−/−*^ mice. Since female *WT*^*+/+*^ mice did not respond to loading in a significant dose:responsive manner, the similar lack of responsiveness of the *Lrp5*^*−/−*^ females could not be ascribed to their Lrp5 status. Cortical bone loss associated with disuse showed no differences between *Lrp5*^*−/−*^ mice and *WT*^*+/+*^ controls, but in cancellous bone of both male and females of these mice, there was a greater loss than in *WT*^*+/+*^ controls. In contrast, the tibias of male and female mice heterozygous for the *Lrp5* G171V HBM mutation showed greater osteogenic responsiveness to loading and less bone loss associated with disuse than their *WT*^*HBM−*^ controls. These data indicate that the presence of the *Lrp5* G171V HBM mutation is associated with an increased osteogenic response to loading but support only a marginal gender-related role for normal Lrp5 function in this loading-related response.

## Research highlights

*► Lrp5*^−/−^ increased cancellous but not cortical bone loss with disuse. *► Lrp5*^−/−^ decreases load-induced new bone formation in males but not in females. *► Lrp5* G171V High Bone Mass mutation protected against bone loss with disuse but increased bone response to loading.

## Introduction

Bone mass and architecture are thought to adapt to be appropriate for the mechanical loading they experience by a mechanism in which load-induced strains, within the bone tissue, influence resident bone cells to control modelling and remodelling to achieve and maintain target levels of strain. The mechanism(s) by which resident bone cells respond to their strain environment is complex and involves the activation of a number of signalling pathways including the canonical Wnt pathway, prostaglandins, nitric oxide, extracellular signal-related kinases and oestrogen receptor-α [1–6].

The involvement of the Wnt pathway in strain-related regulation of bone architecture was predicted from the discovery that two unrelated families of Caucasian origin, with bones of essentially normal appearance but BMD z scores ranging from 4 to 7, had an autosomal dominant mutation mapped to the gene for the low-density lipoprotein receptor-related protein 5 (*Lrp5*) [7,8]. It is through the Lrp5/Frizzled co-receptor that extracellular Wnts activate the Wnt pathway. Discovery of this association between mutation of the *Lrp5* gene and high bone mass occurred around the same time as the realisation that osteoporosis pseudoglioma syndrome (OPPG), a rare autosomal recessive condition characterised by low bone mass, was associated with a loss of function mutation in the same *Lrp5* gene [9]. An explanation for the skeletal phenotype of both these groups could be that the osteoregulatory effects of mechanical strain influence Wnt signalling through the Lrp5 receptor. This explanation envisages the low bone mass in OPPG patients being due to inadequate strain-related stimulation of the Wnt pathway resulting from failure of Wnt stimulation at the Lrp5 receptor [8–10]. The high bone mass (HBM) in people with the *Lrp5* mutation could be explained as being due to an exaggerated response to strain-related stimulation at the same receptor [8,10].

A potential mechanism for this hypothetical link between the osteogenic effects of strain and the Wnt pathway became evident with reports that sclerostin was a ligand for the Lrp5 receptor [11,12]. Sclerostin, the protein product of the *SOST* gene predominately expressed in osteocytes, is down-regulated by high local mechanical strain *in vivo* and *SOST* expression is up-regulated in the absence of loading [3,13]. Thus in normal individuals high strains would act to depress sclerostin production allowing increased activity of the Wnt/Lrp5 pathway and enhanced bone formation. Low strains would be associated with high levels of sclerostin which would down-regulate activity of the Wnt/Lrp5 pathway with subsequent reduced bone formation. This could be one of the ways in which functional strains influence bone mass.

Experiments on mice have shown that animals with the *Lrp5* G171V HBM mutation recapitulate the HBM phenotype found in humans [14]. Those with the *Lrp5* loss of function mutation also have a low bone mass phenotype similar to humans with OPPG [15]. Sawakami et al. (2006) report that the osteogenic response to mechanical load is significantly lower in male and female mice with the *Lrp5* loss of function mutation (*Lrp5*^*−/−*^) compared with Wild Type (*WT*^*+/+*^) controls [16], while Akhter et al. (2004) report that load-induced cortical bone formation is higher in female mice heterozygous for the *Lrp5* Gl71V HBM mutation (*Lrp5^HBM+^*) than in their WT (*WT^HBM−^*) controls [17]. Both of these reports are consistent with the hypothesis that Lrp5/Wnt signalling is involved in the osteoregulatory response of cortical bone to mechanical loading.

Both Sawakami et al. and Akhter et al. performed their experiments using the axially loadable ulna technique originally developed in the rat by Torrance et al. [18] but now routinely applied to the mouse [3,16,19–21]. One disadvantage of using the ulna is that it does not allow examination of loading-related effects on (re)modelling in trabecular bone. Another disadvantage is that it is not easy experimentally to induce disuse in the front limb and to assess the effects of removal of normal functional loading. To avoid both these disadvantages we used the axially loadable tibia technique [21] and applied different load magnitudes to the tibias of male and female mice, either expressing the *Lrp5* G171V HBM mutation [14] or the *Lrp5* loss of function [15] and their respective WT controls. In addition to examining the osteogenic effect of additional loading at different magnitudes we also examined the effect of disuse which we imposed by unilateral sciatic neurectomy. By these means we compared the responses in bones of mice of both genders to 1) the degree of bone loss when functional loading is removed — which could represent the degree of elevation of bone mass from basal (genetically determined) levels due to normal functional loading; and 2) the increment of loading-related new bone stimulated per unit of strain to which they were exposed — which is a measure of their responsiveness to strain. Our hypothesis was that high responsiveness to loading would be associated with increased bone loss due to disuse and a steep “responsiveness” curve between strain magnitude and the increase in new bone formation.

## Materials and methods

### Animal model

Two mouse colonies were used, one which expressed the G171V HBM mutation to the *Lrp5* gene, the other *Lrp5* knock-outs lacking any *Lrp5* activity. Both colonies were generated as previously reported [14,15,22]. The *Lrp5*^*−/−*^ mice were created on a C57BL/6J background by generating an allele that disrupts the extracellular domain of *Lrp5* by inserting an IRES-Lac-Z/Neomycin cassette at amino acid 373 [15]. When correctly targeted, this allele produces no functional *Lrp5* receptor or receptor fragments [15]. To create the *Lrp5*^*−/−*^ mice used in these experiments we interbred mice that were heterozygous for the targeted disruption of *Lrp5* and obtained *WT*^*+/+*^, *Lrp5*^+/−^ and *Lrp5*^*−/−*^ offspring. Genotyping was performed by PCR of DNA obtained from ear biopsies in mice at 3 weeks of age. Wild Type alleles were amplified using primers P1 located in intron 6 (5′-GCCTAGCAAGGGCAGAACAG-3′) and P2 located in intron 7 (5′-CTGGCCTCTGCATGAAACTCT-3′). Mutant alleles were amplified using PCR primers P1 and P3 located in LacZ sequence (5′-TCTTCGCTATTACGCCAGCTG-3′). A 278 base pair fragment was identified in *WT*^*+/+*^ mice and a 200 bp fragment in *Lrp5*^*−/−*^ mice. Both fragments were found in *Lrp5^+/−^* mice.

The *Lrp5^HBM+^* mouse contains two normal copies of the *Lrp5* gene and one copy of the human *Lrp5* gene with the HBM mutation (G171V) linked downstream of a 3.6-kb rat type 1 collagen promoter and integrated into the C57BL/6Tac mouse genome [14]. Babij et al. confirmed the integration and integrity of the transgene using Southern blotting of genomic DNA [14]. In the HBM colony, male *Lrp5*^*HBM+*^ and female F*WT*^*HBM−*^ mice were mated resulting in male and female offspring for the *Lrp5*^*HBM+*^ and *WT*^*HBM−*^ mice. At 3 weeks of age, genotyping of ear snip DNA was performed by PCR using the following forward and reverse primers: 5′-GAA TGG CGC CCC CGA CGA C and 5′-GCT CCC ATT CAT CAG TTC CAT AGG, respectively. *Lrp5^HBM+^* mice showed a 524 bp fragment and *WT*^*HBM−*^ mice did not.

Mice from both colonies were housed up to 5 per cage in polypropylene cages with wood chip and paper bedding and provided standard mouse chow and water *ad libitum* throughout the study. Weaners up to 8 weeks of age were fed a standard rodent breeding diet and thereafter a standard rodent maintenance diet (Special Diet Services, South Witham, UK). All procedures complied with the UK Animals (Scientific Procedures) Act 1986 and were reviewed and approved by the ethics committee of the Royal Veterinary College (London, UK).

### Mechanical strain measurement during dynamic axial loading

**Fig. 1:**
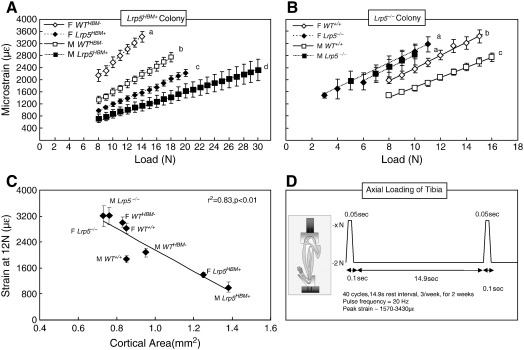
A–B. Strain gauge measurements (με) recorded on the proximal-medial aspect of the tibia (37% of bone length) in the *Lrp5*^*HBM+*^ and *Lrp5*^*−/−*^ colonies. Different letters denote a significant difference in the strains measured between the groups (p < 0.01). Lower strains were associated with a larger cortical area in both male and female *Lrp5*^*HBM+*^ mice; conversely higher strains were associated with a smaller cortical area in male and female *Lrp5*^*−/−*^ mice. The ranking in terms of resistance to strain was M *Lrp5*^*HBM+*^, F *Lrp5*^*HBM+*^, M *WT*^*HBM−*^, M *WT*^*+/+*^, F *WT*^*+/+*^, F *WT*^*HBM−*^, M *Lrp5*^*−/−*^, F *Lrp5*^*−/−*^. C. Relationship between cortical area and strain measured at the same site when 12 N of load was applied during *ex vivo* strain gauging. The strong linear relationship (r^2^ = 0.83, p < 0.01) reflects the higher strains measured in the smaller bones of the *Lrp5*^*−/−*^mice compared with lower strains recorded in the larger bones of the *Lrp5*^*HBM+*^mice. D. Schematic of the loading regime used for the *in vivo* loading experiment. Data shown are the mean ± SE (n = 5/group).

The magnitude of longitudinal mechanical strain at the tibial midshaft resulting from the loads applied to the tibia was established *ex vivo* in a sub-sample of male and female *Lrp5*^*HBM+*^ and *Lrp5*^*−/−*^ mice and their respective WT littermate controls. In each mouse a single element strain gauge (EA-06-015DJ-120, Vishay Measurement Group, NC) was bonded with cyanoacrylate adhesive in longitudinal alignment to the medial aspect of the tibia at 37% of its length from the proximal end. Previous studies have shown that this region corresponds to the site of greatest osteogenic response to similar loading [23]. Strains were measured across a range of peak compressive loads between 8 and 30 N (Figs. 1 A–B). These peak loads were applied with a ramped trapezoidal waveform using the same servo-hydraulic machine (Dartec HC10, Zwick Roell, Herefordshire, UK) used for *in vivo* loading. When the compressive force is applied to the tibia the bone bends in the medial-lateral direction resulting in tension on the medial surface and compression on the lateral surface [24]. From the data in Figs. 1 A–B, three magnitudes of peak load were selected for use in the loading experiment. These were chosen to engender measurable, graded osteogenic responses without causing damage to the bones or joints or the skin through which the loads were applied.

Prior to the *in vivo* loading experiment, strain gauges were used to measure the longitudinal strains applied to the medial surface of the tibia (*ex vivo*) for each group of mice across a range of compressive forces (Figs. 1A–B). The strain engendered at each load (N) was significantly less in the male and female *Lrp5*^*HBM+*^ mice compared with their *WT*^*HBM−*^ littermates (p < 0.01) and significantly greater in the male and female *Lrp5*^*−/−*^ mice compared with their *WT*^+/+^ littermates (p < 0.01). Fig. 1C demonstrates that these strains strongly correlated with the cortical area measured at this site in each group of mice (r^2^ = 0.83, p < 0.01).

### *In vivo* bone loading — the tibia loading protocol

Seventeen week old male and female *Lrp5*^*HBM+*^ or *Lrp5*^*−/−*^ mice and their respective WT littermates were randomly assigned to one of three loading groups (n = 8/group). While under oxygen and halothane anaesthetic (Merial, Ireland) the right tibia from each mouse was axially loaded on 3 alternate days per week for 2 weeks for 40 cycles/day with a trapezoid waveform, with 14.9 second rest between cycles (Fig. 1D). The left tibia was used as a non-loaded control to allow side-to-side comparisons for the effects of loading on (re)modelling. The use of the contra-lateral limb as a control using this protocol has been validated in our laboratory by comparing (re)modelling in the bones of limbs contra-lateral to those used in loading experiments with that in normal limbs of separate animals to which no loads had been applied [25]. All mice were allowed normal cage activity in between loading sessions. At 19 weeks of age, the mice were euthanized and their tibias dissected free of soft tissue and stored in 70% ethanol.

### Reduced bone loading — sciatic neurectomy

At 14 weeks of age, female and male *Lrp5*^*HBM+*^, *Lrp5*^*−/−*^, *WT*^*HBM−*^ and *WT^+/+^* mice (n = 6 to 9) underwent unilateral sciatic neurectomy to remove functional load bearing of the right tibia [26]. The mice were anaesthetised using halothane and oxygen, the sciatic nerve approached from its dorsal surface and a 3 mm section excised. The wound was sutured and the mice recovered in a heated cage. The left tibia served as a control. Three weeks after neurectomy the mice were euthanized and the right and left tibia were extracted and stored in 70% ethanol until they were scanned using microCT.

### MicroCT

The entire tibias from loaded and sciatic neurectomised groups were scanned *ex-vivo* at a resolution of 4.9 μm × 4.9 μm using micro computed tomography (Skyscan 1172, Belgium). Analysis of cortical bone was performed using a 0.49 mm long segment (or 100 tomograms) at 37% of the tibias' length from their proximal ends. This was the site where the strain gauges were attached and where previous experiments had established a substantial osteogenic response to loading [23]. For analysis of the cortical bone compartment, 2D computation was used and parameters determined for each of the 100 tomograms which were then averaged. The parameters chosen for cortical bone were: total (periosteally enclosed) area, medullary (endosteally enclosed) area and cortical bone area (total–medullary). For trabecular bone, we analysed a region of secondary spongiosa located distal to the growth plate in the proximal metaphysis and extending 0.98 mm (or 200 tomograms) distally. Woven bone was detected in less than 10% of all loaded mice. Histomorphometric analysis in 2- and 3-dimensions (2D, 3D) was performed by Skyscan software (CT-Analyser v.1.5.1.3). For analysis of cancellous bone the cortical shell was excluded by operator-drawn regions of interest and 3D algorithms used to determine: bone volume percentage (BV/TV), trabecular thickness (Tb.Th), trabecular number (Tb.N) and trabecular spacing (Tb.Sp). Coefficients of variation (CVs) were determined by repeating full scan (including repositioning) reconstruction and analysing the same sample 4 times. The CV of each parameter was determined as the ratio between the standard deviation and the mean. The CVs for relevant parameters are the following: BV/TV: 1.57% and Tb.Th: 1.61% and cortical area: 0.11%.

### Statistical analysis

An analysis of variance (ANOVA) was used to determine the main effects of gender and genotype and any interaction between these on all phenotypic measurements and on the percent side-to-side difference between the right sciatic neurectomised and left control limbs ((R − L) / L * 100). When a significance value of < 0.05 was detected a Bonferroni post-hoc analysis was undertaken. All data were analysed using SPSS for Windows, Version 16.0 (SPSS Inc., Chicago, IL, USA).

To assess the effects of sciatic neurectomy and loading, paired sample t-tests were conducted on the treated vs. the non-treated control limbs. Un-paired t-tests were performed on the percent change due to loading between *Lrp5*^*HBM+*^/*Lrp5*^*−/−*^ mice and their WT littermates at similar magnitudes of strain. For each genotype we then compared the bone changes in response to the three magnitudes of load applied *in vivo.* We did this by plotting the percent side-to-side difference in the loaded vs. non-loaded limbs at their corresponding strain. We could find no curve that fitted these data better than a straight line and so for the purposes of analysis we proceeded on that basis. For each group of mice we used an ANCOVA using strain as a covariate to establish the presence of significant genotype:strain interactions. When these were detected we ran a contrast analysis in SAS for Windows Version 9.2 (SAS Institute Inc, Cary, NC, USA) to establish whether the slope of the strain:response line was significantly different from zero (indicating a statistically significant dose:response) and whether it was significantly different from that in the other groups. All tests were considered significant at p < 0.05.

## Results

### Basal phenotype

The phenotype of the male and female mice from *Lrp5*^*HBM+*^ and *Lrp5*^*−/−*^ colonies and their respective WT littermate controls were similar to those previously reported [14,15]. In summary, there was no significant difference in tibial bone length or body weight between the *WT*^*HBM−*^ and *Lrp5*^*HBM+*^ mice, but all measures of cortical and cancellous bone, except Tb.Sp, were higher in the *Lrp5*^*HBM+*^ animals than their *WT*^*HBM−*^ controls. In the *Lrp5*^*−/−*^ colony, body weight was significantly greater in *WT*^*+/+*^ mice than *Lrp5*^*−/−*^ mice but there was no difference in tibial length. All cortical bone parameters, except medullary area, and all measures of cancellous bone, except Tb.Sp, were lower in the *Lrp5*^*−/−*^ animals than their *WT*^*+/+*^ controls. Interestingly, animals from the *WT*^*+/+*^ background have a slightly more robust cortical bone phenotype than those of the *WT*^*HBM−*^, whereas *WT*^*HBM−*^ have a more robust trabecular bone phenotype than those of the *WT*^*+/+*^.

### The response to disuse

From Table 1 it can be seen that gender had a significant effect on the magnitude of change in cortical area and total area in response to sciatic neurectomy. Female mice lost more cortical bone than male mice (− 12.5% vs. − 8.3%, respectively, p < 0.001, data not shown) due to a greater reduction in total area. Genotype also had an effect on change in cortical area, with the *Lrp5*^*HBM+*^ mice losing less bone than all other genotypes (− 5.5% vs., − 14.4% WT^HBM−^, − 10.4% WT^+/+^, − 11.4% Lrp5^−/−^, p < 0.05, data not shown). In contrast, no difference in cortical bone loss was detected between *Lrp5*^*−/−*^ mice and their *WT*^*+/+*^ littermates. A three-way interaction between gender, genotype and sciatic neurectomy was only detected for medullary area. The post-hoc analysis showed that female *Lrp5*^*HBM+*^ mice experienced less endocortical expansion than female *WT*^*HBM−*^ mice (medullary area: 6.3 ± 3.8% vs. 16.4 ± 2.2% respectively, p < 0.05), no other differences were detected between male *Lrp5*^*HBM+*^ and their *WT*^*HBM−*^ littermates or between male and female *Lrp5*^*−/−*^ mice and their *WT*^*+/+*^ littermates.

**Fig. 2:**
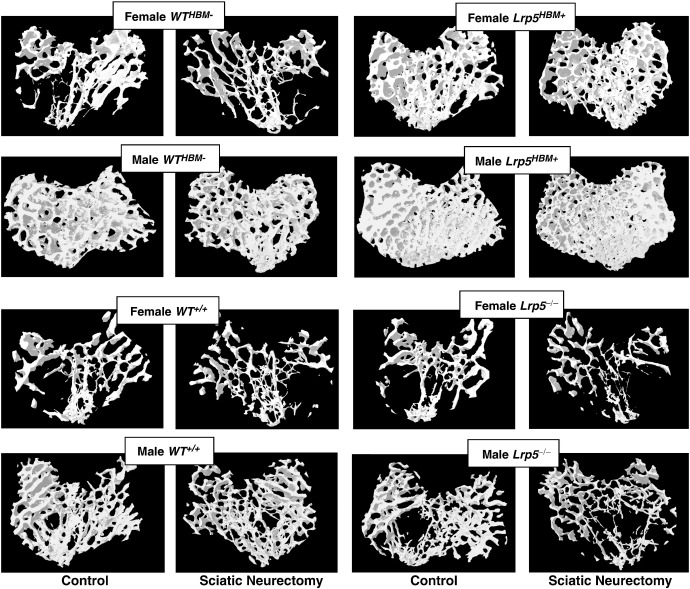
Representative microCT images of trabecular bone in the sciatic neurectomised (right) and contra-lateral normally loaded control tibias (left) from each of the 8 groups of mice.

In cancellous bone, gender had a significant effect on the magnitude of sciatic neurectomy-induced change in Tb.Th and Tb.N, but not BV/TV or Tb.Sp, with male mice losing slightly more Tb.Th (− 20.2% vs. − 16.7%, respectively, p < 0.05, data not shown) and females losing more Tb.N (− 24.9% vs. − 22.9%, respectively, p < 0.05, data not shown). Genotype also had a significant effect on the magnitude of loss on all parameters of cancellous bone. *Lrp5*^*HBM+*^ mice experienced less loss in BV/TV than their *WT*^*HBM−*^ littermates (− 17.2% vs. − 43.3%, respectively, p < 0.05, data not shown). This could be attributed to a reduced loss in Tb.Th and Tb.N. In contrast, *Lrp5*^*−/−*^ mice showed a greater loss in BV/TV than their *WT*^*+/+*^ littermates (− 52.4% vs. − 41.3% respectively, p < 0.05, data not shown) due to a greater reduction in Tb.N and increase in Tb.Sp. A three-way interaction between gender, genotype and sciatic neurectomy was not detected for any of the cancellous bone parameters; therefore bone loss was similar in male and female mice within each genotype. The trabecular architecture in the control and sciatic neurectomised limbs of the eight groups of mice are illustrated in Fig. 2.

In summary these findings show that the degree of cortical and cancellous bone loss associated with sciatic neurectomy is affected by *Lrp5* status. The presence of the *Lrp5* HBM mutation is associated with less loss in cortical and cancellous bone than in their *WT*^*HBM−*^ controls. The lack of difference in cortical bone loss with disuse between *Lrp5*^*−/−*^ mice and their *WT*^*+/+*^ controls indicates that normal *Lrp5* function has no effect on this process. However, in cancellous bone absence of *Lrp5* is associated with a greater decrease in Tb.N and increase in Tb.Sp than in *WT*^*+/+*^ controls.

### Sensitivity to graded mechanical loading *in vivo* in *Lrp5*^*−/−*^ mice

**Fig. 3:**
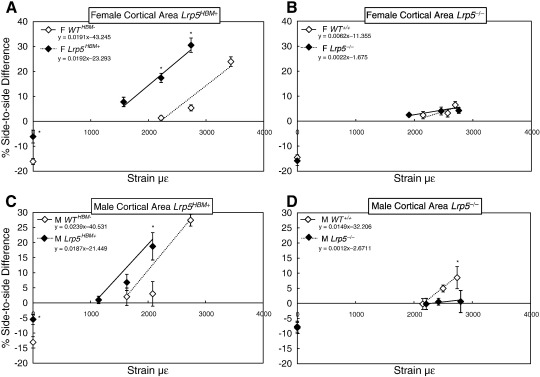
Percent difference in cortical area between right (treated) and left (control) limbs in response to disuse induced by sciatic neurectomy (0 με) and varying magnitudes of strain during *in vivo* axial loading in female and male mice with the *Lrp5*^*HBM+*^ and *WT*^*HBM−*^ (A and C), and the *Lrp5*^*−/−*^ and *WT*^*+/+*^ (B and D) backgrounds. The plotted line only includes the loading data and the equations of these lines are provided. Significant strain:genotype interactions indicated that the slopes were different between the genotypes (see Tables 3 and 4). Unpaired sample t-tests compared the percent change in *Lrp5*^*HBM+*^ and *Lrp5*^*−/−*^ with their WT littermates at similar magnitudes of strain. Data shown are the mean ± SE (n = 8/group). *p < 0.05 to < 0.001.

Mechanical loading significantly and dose-responsively increased the cortical bone parameters, % cortical bone area and % total area in *WT*^*+/+*^ males, but *Lrp5*^*−/−*^ males showed a complete absence of cortical bone responses (Table 2, Fig. 3). Female *WT*^*+/+*^ mice failed to respond dose-responsively to loading for cortical bone parameters (Table 3), but some of the individual load groups produced significant side-to-side loading effects for cortical variables (Table 2). Like their WT counterparts, *Lrp5*^*−/−*^ females showed no dose–response to loading in cortical parameters, but significant side-to-side loading effects for some cortical bone parameters were found (Table 2 Fig. 3). Half of the significant side-to-side loading effects found in *Lrp5*^*−/−*^ females were found in outcomes/strains that did not engender effects in the female *WT*^*+/+*^ mice, which complicated the interpretation.

Trabecular bone analysis of loading effects in the same mice showed that of the four trabecular bone parameters analysed, only Tb.Th increased dose responsively in the male *WT*^*+/+*^ mice (Table 4). Tb.Th in the male *Lrp5*^*−/−*^ counterparts did not show a dose–response with loading, though analysis of the side-to-side differences showed modest but significant Tb.Th loading effects at all 3 load levels in *Lrp5*^*−/−*^ males (Table 2). The magnitude of this response in Tb.Th was similar to that found in male *WT^+/+^* mice.

**Fig. 4:**
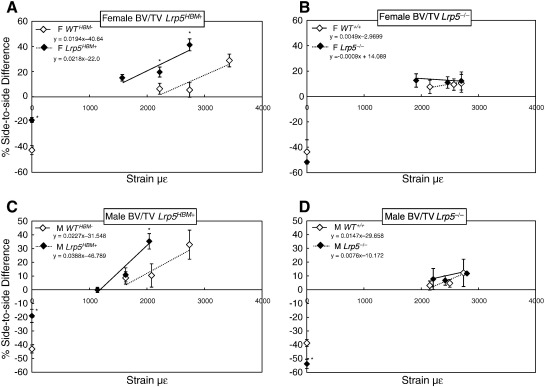
Percent difference in BV/TV between right (treated) and left (control) limbs in response to disuse induced by sciatic neurectomy (0 με) and varying magnitudes of strain during *in vivo* axial loading in female and male mice with the *Lrp5*^*HBM+*^ and *WT*^*HBM−*^ (A and C), and the *Lrp5*^*−/−*^ and *WT*^*+/+*^ (B and D) backgrounds. The plotted line only includes the loading data and the equations of these lines are provided. Significant strain:genotype interactions denoted a difference in the slopes between the genotypes (see Tables 3 and 4). Unpaired sample t-tests compared the percent change in *Lrp5*^*HBM+*^ and *Lrp5*^*−/−*^ with their WT littermates at similar magnitudes of strain. Data shown are the mean ± SE (n = 8/group). *p < 0.05 to < 0.001.

Female *WT*^*+/+*^ and *Lrp5*^*−/−*^ mice did not respond dose-responsively to any of the trabecular parameters, the one exception being Tb.Th in *Lrp5*^*−/−*^ mice (Table 3, Fig. 4). However, since the female *WT*^*+/+*^ mice did not respond to loading in a significant dose:responsive manner, the effect in Tb.Th is difficult to interpret. Among the *WT*^*+/+*^ females, Tb.Th in the high load group was the only outcome that produced a significant side-to-side effect (Table 2). Female *Lrp5*^*−/−*^ showed significant side-to-side loading effects in BV/TV at the medium load, and in Tb.Th in the medium and high loads, but interpretation of this effect is difficult because the *WT*^*+/+*^ controls did not respond for one of the three effects found in *Lrp5*^*−/−*^ females.

### Sensitivity to graded mechanical loading *in vivo* in *Lrp5*^*HBM+*^ mice

Mechanical loading significantly and dose-responsively increased the cortical bone parameters, % cortical bone area and % total area in *WT*^*HBM−*^ and *Lrp5*^*HBM+*^ male and female mice (Fig. 3, Tables 3 and 4). A significant dose-responsive reduction in medullary area was observed in *Lrp5*^*HBM+*^ females, but not in their WT controls (Table 3). Analysis of side-to-side differences at individual strain levels indicate that the *Lrp5*^*HBM+*^ mice respond significantly at strains insufficient to induce a similar cortical response in *WT*^*HBM−*^ mice, and when *WT*^*HBM−*^ mice do show a significant side-to-side effect, the *Lrp5*^*HBM+*^ response is typically significantly greater (Table 2, Fig. 3).

Trabecular bone analysis of loading effects in the same mice showed that mechanical loading significantly and dose-responsively increased BV/TV and Tb.Th in male and female *WT*^*HBM−*^ and *Lrp5*^*HBM+*^ mice (Fig. 4, Tables 3 and 4). Post-hoc analysis of the strain:response slopes indicated that the Tb.Th response to loading was significantly enhanced in male and female *Lrp5*^*HBM+*^ mice, compared with their respective *WT*^*HBM−*^ controls. Analysis of side-to-side differences at individual strain levels indicate that the *Lrp5*^*HBM+*^ mice respond significantly at strains insufficient to induce similar trabecular responses in *WT*^*HBM−*^ mice, and when *WT*^*HBM−*^ mice do show a significant side-to-side effect, the *Lrp5*^*HBM+*^ response is typically significantly greater (Table 2).

## Discussion

The primary objective of the experiments described in this paper was to establish the role of *Lrp5* in bone's response to mechanical loading. We did this by comparing the tibial response to increased magnitudes of strain, and to disuse, in males and females from two groups of mice: those that lack a functional *Lrp5* receptor (*Lrp5*^*−/−*^) and those that express the human *Lrp5* G171V gain of function mutation (*Lrp5*^*HBM+*^). The overall inferences from the study are that lack of *Lrp5* function i) has no influence on the amount of disuse-related bone loss in cortical bone but is associated with greater bone loss in cancellous bone; and ii) prevents load-induced bone formation in the cortex and inhibits the response in trabecular bone in male mice. It is difficult to conclude whether *Lrp5* status had similar effects in female mice since for most parameters, neither the female *Lrp5*^*−/−*^ mice nor their *WT*^*+/+*^ littermate controls showed a significant dose:response to loading. In contrast, the presence of the *Lrp5* G171V HBM mutation in both males and females was associated with some protection against disuse-related bone loss in both cortical and cancellous bone and an increased osteogenic responsiveness to loading that was especially apparent in the females.

The rationale for examining the bone loss associated with disuse in these groups of mice was our hypothesis that if a more robust skeletal phenotype is a result of greater responsiveness to loading then the degree of bone loss associated with removal of the loading-related stimulus should also be greater. Conversely if a less robust skeletal phenotype were to be due to a lower osteogenic responsiveness to loading this should be reflected by a lower level of bone loss associated with disuse. In this experiment a direct comparison between all the genders and genotypes investigated was complicated by basal differences between the WT background of the *Lrp5*^*HBM+*^ and *Lrp5*^*−/−*^ colonies. This may have effects outside and in addition to anything related to loading. It is unknown whether osteoclast activity (which in these almost mature animals would have been responsible for the lower bone mass associated with disuse) is similar in timing or extent in the different groups, even though it has been shown that *Lrp5*^*HBM+*^ and *Lrp5*^*−/−*^ mice show no differences in their osteoclast number compared with WT controls [14,15]. With these reservations in mind, but assuming that such differences between groups are minor compared with the main effects of their *Lrp5* genotype, the outcome of the disuse experiment appears to be that in cortical bone the degree of bone loss is unaffected by the absence of functional *Lrp5*. In cancellous bone, absence of a functional *Lrp5* receptor is associated with greater disuse-related increase in trabecular spacing and decrease in BV/TV and trabecular number than in WT controls. In contrast the presence of the *Lrp5* G171V HBM mutation in the *Lrp5*^*HBM+*^ mice is associated with less loss of cortical and trabecular bone than in their *WT*^*HBM−*^ controls. Similar findings on *Lrp5*^*HBM+*^ and *Lrp5*^*−/−*^ mice were reported by Bex et al. and Akhter et al. [27,28]. This suggests that the more robust phenotype of these animals may not be solely due to any enhanced osteogenic response to loading but includes additional osteogenic responses to other stimuli with osteoregulatory effects.

The rationale for examining the osteogenic response to loading was to assess more directly the potential role of *Lrp5* in bone's responsiveness to mechanical loading. Since male *Lrp5*^*−/−*^ mice show no osteogenic response to loading in the bone cortex, and an absence of dose-responsiveness for trabecular thickness, compared with their dose-responsive WT controls, this could be ascribed to their *Lrp5* status. In contrast, female *Lrp5*^*−/−*^ mice showed a similar percent increase in cortical area and cancellous bone as their *WT*^*+/+*^ littermates in response to high strains (Fig. 3). Interpretation of how the absence of *Lrp5* influences the sensitivity across a range of strains is problematic in the female mice given that the female WT^+/+^ mice of the *Lrp5*^*−/−*^ colony did not themselves show a dose:response relationship. The possibility that this lack of response was due to the magnitude of the peak strains not reaching an appropriate threshold is unlikely since at each strain there was a significant osteogenic response to loading that increased incrementally with strain. This was, however, not enough to show a significant a dose:response relationship. Similarly the possibility that there was some experimental error in the loading of this cohort of mice is unlikely since they were loaded and analysed as a mixed population interspersed within the other groups. Thus, while we cannot explain the apparently anomalous absence of a statistically significant dose:response relationship with increasing strain we have at present no grounds on which to discount it being a real phenomenon.

All mice from the *Lrp5*^*HBM+*^ colony showed a significant dose:response to loading in both cortical and cancellous bone parameters. The magnitude of this response was greater in mice expressing the *Lrp5* gain of function mutation. There also appeared to be an influence of gender on the sensitivity of these mice to loading. Female mice with the *Lrp5*^*HBM+*^ genotype show a significant osteogenic response at much lower magnitudes of strain than all other male and female genotypes. Although this is of interest, we have no evidence for the mechanism involved. Furthermore, the reason for the increased sensitivity to loading and reduced bone loss in response to disuse in *Lrp5*^*HBM+*^ mice is not clear. While it is tempting to ascribe this solely to the presence and activity of the *Lrp5* G171V HBM mutation, we do not know if increased sensitivity to loading is a function of the specific activity of the mutation or the result of having 4-fold higher *Lrp5* mRNA expression in bone compared with controls [14]. There is support for it being the latter since Babij et al. [14] have shown that over expression of the wild type, non-mutated, *Lrp5* gene in bone of mice results in a modest increase in bone mass.

The results from our present study need to be compared with those of previous studies with similar objectives. Akhter et al. [17] report that in cortical bone female mice with the *Lrp5*^*HBM+*^ genotype showed greater increases in periosteal bone formation rates than WT controls in response to 5 days of tibial four-point bending. The preliminary data from Hackfort et al., who axially loaded the tibia of female *Lrp5*^*−/−*^ mice [29], suggest that the absence of *Lrp5* has no effect on the responsiveness of cortical bone to mechanical loading. These latter results are inconsistent with the data we generated for male mice, though the comparison to our female data is inconclusive. Our findings on male *Lrp5*^*−/−*^ mice are consistent with the findings of Sawakami et al. who report that after 3 days of sequential loading of the ulna, male and female *Lrp5*^*−/−*^ mice show an 88 to 99% lower response to loading in the cortical bone than WT controls [16].

Sawakami et al. also reported that male and female *Lrp5*^*−/−*^ mice are equally capable as *WT*^*+/+*^ mice at recruiting osteoblasts in response to a single period of mechanical loading and that absence of functional *Lrp5* had little effect on early mediators of mechanical signalling, such as ATP and PGE2 release or ERK1/2 activation, that are detectable within seconds or minutes of mechanical stimulation. They attributed the deficiency of the fully osteogenic adaptive responses in their study to the inability of *Lrp5*^*−/−*^ osteoblasts to synthesise the bone matrix protein osteopontin. This would explain the significantly reduced osteogenic response in male *Lrp5*^*−/−*^ mice and supports the notion that the canonical Wnt signalling has a role in bone cells' response to mechanical loading. However, other data suggest that the mechanism might not be so clear cut as indicated by Kato's finding that Wnt-signalling still partially occurs in osteoblasts from *Lrp5*^*−/−*^ mice [15], by Robling's finding that the sclerostin antibody can improve bone mass whether *Lrp5* is present or not [30], or by the *in vitro* findings by Sunters et al. [31] and Case et al. [1] showing that during the early phase of the strain response, activation of the chief effector of the canonical Wnt pathway (β-catenin) is not contingent on Wnts interacting with the Lrp5 receptor. Thus, the required post-loading pathways in bone cells may also depend on other receptors, possibly Lrp4 [32] or Lrp6 [2].

The data we present here, at least in male mice, are consistent with the differences in bone mass between normal WT mice and those that lack *Lrp5* function, being due to an altered responsiveness to bone loading. Karsenty and colleagues attribute the low bone mass of the *Lrp5*^*−/−*^ related phenotype to the effect of *Lrp5* on serotonin secretion in the duodenum [33]. However, this finding has not been replicated [34]. The *Lrp5*^*−/−*^ mice in our study, as in that of Karsenty and colleagues, may have had high serotonin levels. However, Warden et al. [35] suggest that these high serotonin levels would not themselves affect the bones' adaptive responses to loading. Our data are therefore not inconsistent with Karsenty's conclusion but neither do they support it.

In conclusion, the data presented here indicate that the expression of the human *Lrp5* G171V HBM mutation is associated in both cortical and cancellous bone with an increased osteogenic responsiveness to supra-physiological loading, which is more marked in females than males, and with some protection against the bone loss associated with neurectomy-induced disuse. Absence of normal *Lrp5* activity is associated in both males and females with greater neurectomy-induced bone loss in cancellous bone than in WT controls but there is no difference between these genotypes in the level of bone loss in the cortex. Absence of *Lrp5* activity abolished the percent increase in cortical bone gain in response to loading in males but similar experiments in females showing no difference in loading-related response between those with and without functional *Lrp5* were inconclusive since for most parameters neither the female *Lrp5*^*−/−*^ mice nor their *WT*^*+/+*^ littermate controls, showed a statistically significant dose:response to loading.

## Figures and Tables

**Table 1:** Bone parameters of the tibia in control (C) limbs and the percent change in the sciatic neurectomised limbs (SN) ((SN − C) / C * 100) of all male and female *WT*^*HBM−*^, *Lrp5*^*HBM+*^, *WT*^*+/+*^ and *Lrp5*^−/−^ mice. Data shown are the mean ± SE. Paired t-tests were performed on the sciatic neurectomised vs. the control limbs; significant differences are shown in bold (p < 0.05 to < 0.001). ANOVA was performed on the percent changes between the control and sciatic neurectomised limbs.

	F *WT*^*HBM−*^	F *Lrp5*^*HBM+*^	F *WT*^*+/+*^	F *Lrp5*^*−/−*^	M *WT*^*HBM−*^	M *Lrp5*^*HBM+*^	M *WT*^*+/+*^	M *Lrp5*^*−/−*^	Gender	Genotype	Gender* genotype
	n = 7	n = 9	n = 8	n = 7	n = 9	n = 8	n = 8	n = 8			
*Cortical bone*											
Cortical area (mm^2^) C	0.831 ± 0.020	1.206 ± 0.034	0.846 ± 0.006	0.734 ± 0.015	0.876 ± 0.022	1.398 ± 0.048	0.946 ± 0.033	0.739 ± 0.012			
% change SN	**− 16.1 ± 1.2%**	**− 6.1 ± 2.6%**	**− 14.5 ± 1.0%**	**− 15.8 ± 1.9%**	**− 13.1 ± 1.6%**	**− 5.5 ± 1.9%**	**− 7.7 ± 1.8%**	**− 8.1 ± 1.5%**	< 0.001	< 0.001	NS
Total area (mm^2^) C	1.415 ± 0.049	2.000 ± 0.041	1.267 ± 0.015	1.157 ± 0.027	1.582 ± 0.059	2.236 ± 0.048	1.474 ± 0.045	1.205 ± 0.012			
% change SN	− 2.7 ± 1.6%	− 1.3 ± 1.0%	**− 2.6 ± 0.9%**	**− 4.5 ± 1.3%**	**− 3.2 ± 0.9%**	0.0 ± 0.8%	− 1.2 ± 1.8%	0.5 ± 1.0%	< 0.05	NS	NS
Medullary area (mm^2^) C	0.584 ± 0.039	0.791 ± 0.028	0.421 ± 0.012	0.544 ± 0.124	0.706 ± 0.039	0.838 ± 0.021	0.527 ± 0.021	0.456 ± 0.014			
% change SN	**16.4 ± 2.2%**	6.3 ± 3.8%	**21.4 ± 2.5%**	**9.4 ± 3.5%**	**9.1 ± 3.0%**	**9.3 ± 3.0%**	**10.6 ± 2.3%**	**16.6 ± 1.7%**	NS	NS	< 0.05

*Cancellous bone*											
BV/TV (%) C	10.817 ± 0.872	28.556 ± 1.145	8.813 ± 0.409	7.607 ± 0.512	18.598 ± 1.456	34.605 ± 1.742	16.525 ± 1.435	11.665 ± 0.746			
% change SN	**− 42.4 ± 3.5%**	**− 18.5 ± 2.0%**	**− 43.7 ± 4.2%**	**− 51.7 ± 9.6%**	**− 43.1 ± 2.9%**	**− 19.0 ± 4.8%**	**− 38.7 ± 2.5%**	**− 53.8 ± 3.3%**	NS	< 0.001	NS
Tb.Th (mm) C	0.054 ± 0.002	0.067 ± 0.001	0.057 ± 0.001	0.052 ± 0.001	0.053 ± 0.001	0.064 ± 0.002	0.054 ± 0.001	0.050 ± 0.001			
% change SN	**− 22.2 ± 1.5%**	**− 14.9 ± 1.1%**	**− 15.8 ± 2.1%**	**− 11.5 ± 1.2%**	**− 26.4 ± 1.4%**	**− 18.9 ± 3.5%**	**− 20.3 ± 1.4%**	**− 18.3 ± 1.7%**	< 0.05	< 0.001	NS
Tb.N (mm^− 1^) C	2.077 ± 0.283	4.268 ± 0.124	1.547 ± 0.084	1.452 ± 0.083	3.947 ± 0.257	5.350 ± 0.142	3.052 ± 0.276	2.308 ± 0.101			
% change SN	**− 26.3 ± 3.9%**	**− 5.0 ± 2.1%**	**− 32.0 ± 5.1%**	**− 44.8 ± 10.0%**	**− 22.3 ± 2.8%**	− 0.1 ± 2.5%	**− 23.2 ± 2.9%**	**− 43.6 ± 3.5%**	< 0.05	< 0.05	NS
Tb.Sp (mm) C	0.293 ± 0.025	0.166 ± 0.003	0.358 ± 0.018	0.437 ± 0.023	0.181 ± 0.009	0.124 ± 0.003	0.124 ± 0.003	0.312 ± 0.016			
% change SN	1.7 ± 3.0%	**4.8 ± 1.2%**	2.8 ± 5.5%	14.4 ± 9.9%	**13.3 ± 2.9%**	**5.6 ± 1.9%**	**7.4 ± 1.4%**	**20.8 ± 6.7%**	NS	< 0.001	NS

**Table 2:** Percent change in the response to loading in all 8 groups of mice at their corresponding magnitudes of strain (mean ± SE, n = 8/group). Paired t-tests were performed on the loaded vs. the non-loaded limbs; significant differences are shown in bold (p < 0.05 to < 0.001). Unpaired t-tests were conducted on the percent change in *Lrp5*^*HBM*^ and *Lrp5*^*−/−*^ mice compared with their WT littermates at similar magnitudes of strain. * p < 0.05 to < 0.001.

	F *WT*^*HBM−*^	F *Lrp5*^*HBM+*^	F *WT*^*+/+*^	F *Lrp5*^*−/−*^	M *WT*^*HBM−*^	M *Lrp5*^*HBM+*^	M *WT*^*+/+*^	M *Lrp5*^*−/−*^
*Cortical bone*								
Very low strain		1570 ± 100 με (14 N)				1140 ± 160 με (14 N)		
Cortical area %		**7.8 ± 1.9**				1.0 ± 2.9		
Total area %		**4.8 ± 0.6**				0.1 ± 2.4		
Medullary area %		0.4 ± 2.5				− 1.1 ± 2.9		
Low strain	2220 ± 160 με (9 N)	2220 ± 100 με (19.8 N)	2150 ± 120 με (9 N)	1910 ± 250 με (6 N)	1630 ± 120 με (11.5 N)	1630 ± 330 με (19.8 N)	2150 ± 100 με(12.5 N)	2220 ± 280 με (6 N)
Cortical area %	1.4 ± 0.9	17.4 ± 1.8*	2.4 ± 1.4	**2.5 ± 0.5**	2.0 ± 1.2	6.8 ± 4.1	0.2 ± 1.8	− 0.2 ± 1.9
Total area %	2.2 ± 1.4	9.4 ± 1.4*	**4.6 ± 1.2**	1.9 ± 1.0	0.2 ± 1.4	3.0 ± 2.2	0.1 ± 1.5	− 0.7 ± 0.7
Medullary area %	3.4 ± 2.7	− 4.4 ± 1.6*	**9.8 ± 3.8**	1.1 ± 2.3	− 2.2 ± 2.3	− 2.3 ± 3.8	0.7 ± 3.0	− 1.3 ± 1.5
Medium strain	2740 ± 160 με (10.5 N)	2740 ± 150 με (25 N)	2570 ± 150 με (11 N)	2460 ± 210 με (8 N)	2080 ± 130 με (14.5 N)	2080 ± 360 με (26 N)	2500 ± 120 με (14.5 N)	2420 ± 360 με (8 N)
Cortical area %	**5.4 ± 1.2**	30.5 ± 2.9*	3.2 ± 1.5	**4.1 ± 1.6**	3.0 ± 2.7	18.7 ± 1.9*	**4.9 ± 1.1**	0.5 ± 1.7*
Total area %	**5.6 ± 0.9**	14.9 ± 1.0*	**4.5 ± 1.2**	**3.7 ± 1.0**	**3.4 ± 1.4**	8.5 ± 1.5*	**5.6 ± 1.1**	2.1 ± 1.7
Medullary area %	**5.8 ± 1.4**	− 9.8 ± 2.4*	**7.2 ± 2.1**	3.4 ± 2.2	4.0 ± 3.0	− 8.0 ± 1.7*	**7.2 ± 3.1**	4.6 ± 2.4
High strain	3430 ± 180 με (14 N)		2670 ± 160 με (12 N)	2670 ± 130 με (9 N)	2740 ± 160 με (18 N)		2740 ± 140 με (16 N)	2800 ± 320 με (10 N)
Cortical area %	**23.9 ± 1.9**		**6.5 ± 1.3**	**4.2 ± 1.1**	**27.4 ± 4.5**		**8.5 ± 3.6**	0.6 ± 1.2*
Total area %	**16.7 ± 2.0**		**6.0 ± 1.9**	**5.2 ± 0.9**	**12.5 ± 1.9**		**5.6 ± 2.1**	1.6 ± 1.0
Medullary area %	6.5 ± 5.5		5.1 ± 3.2	**7.0 ± 1.5**	− 6.7 ± 3.1		− 1.0 ± 1.5	**3.1 ± 1.3**

*Cancellous bone*								
Very low strain		1570 ± 100 με (14 N)				1140 ± 160 με (14 N)		
BV/TV %		**15.1 ± 2.6**				− 0.1 ± 1.9		
Tb.Th %		**8.9 ± 1.2**				0.9 ± 2.6		
Tb.N %		**2.3 ± 4.6**				− 0.8 ± 1.8		
Low strain	2220 ± 160 με (9 N)	2220 ± 100 με (19.8 N)	2150 ± 120 με (9 N)	1910 ± 250 με (6 N)	1630 ± 120 με (11.5)	1630 ± 330 με (19.8 N)	2150 ± 100 με (12.5 N)	2220 ± 280 με (6 N)
BV/TV %	6.4 ± 4.4	19.7 ± 4.0*	7.6 ± 6.1	2.6 ± 5.3	8.6 ± 4.5	10.9 ± 5.1	3.1 ± 3.3	7.8 ± 7.6
Tb.Th %	4.3 ± 2.0	18.5 ± 2.7*	6.1 ± 2.8	2.0 ± 2.6	6.2 ± 3.2	**11.1 ± 3.8**	**6.1 ± 1.7**	**4.2 ± 1.3**
Tb.N %	2.3 ± 4.6	0.9 ± 2.0	1.3 ± 5.2	10.5 ± 5.0	2.1 ± 1.4	− 0.4 ± 2.0	− 3.8 ± 4.4	3.9 ± 6.4
Medium strain	2740 ± 160 με (10.5 N)	2740 ± 150 με (25 N)	2570 ± 150 με (11 N)	2460 ± 210 με (8 N)	2080 ± 130 με (14.5 N)	2080 ± 360 με (26 N)	2500 ± 120 με (14.5 N)	2420 ± 360 με (8 N)
BV/TV %	5.4 ± 5.8	41.3 ± 4.8*	9.4 ± 5.3	**11.0 ± 4.5**	10.4 ± 8.5	35.3 ± 5.6*	4.7 ± 2.9	6.7 ± 3.5
Tb.Th %	3.3 ± 3.1	36.6 ± 2.8*	**6.3 ± 1.2**	**7.7 ± 2.1**	6.8 ± 5.0	34.2 ± 4.7*	**9.0 ± 3.5**	**4.5 ± 1.0**
Tb.N %	1.6 ± 3.3	3.5 ± 3.0	0.3 ± 3.9	3.4 ± 4.7	2.8 ± 5.1	0.7 ± 0.9	− 3.9 ± 2.9	5.7 ± 2.2
High strain	3430 ± 180 με (14 N)		2670 ± 160 με (12 N)	2670 ± 130 με (9 N)	2740 ± 160 με (18 N)		2740 ± 140 με (16 N)	2800 ± 320 με (10 N)
BV/TV %	**28.9 ± 5.1**		10.4 ± 6.5	12.2 ± 7.1	**32.8 ± 10.6**		12.2 ± 9.9	**11.8 ± 1.0**
Tb.Th %	**20.8 ± 2.9**		**10.5 ± 2.7**	**9.7 ± 2.6**	**25.2 ± 5.4**		**18.4 ± 4.3**	5.6 ± 0.6*
Tb.N %	**6.6 ± 3.2**		0.0 ± 5.5	2.1 ± 5.3	5.4 ± 4.3		− 6.4 ± 5.9	6.1 ± 1.4

**Table 3:** The slope of the strain:response curves representing the responsiveness of each bone parameter to increasing mechanical strains in female mice (mean ± SE, n = 8/group) in response to loading. A significant interaction between the strain and genotype was detected for all cortical bone parameters and trabecular thickness indicating an effect of genotype on gradients of the slope (p < 0.05 to < 0.001). Different letters in a row denotes the groups are significantly different to each other, as detected from the post-hoc analysis (p < 0.05 to < 0.001). Slopes in bold are significantly different from zero.

	F *WT*^*HBM−*^	F *Lrp5*^*HBM+*^	F *WT*^*+/+*^	F *Lrp5*^*−/−*^
*Cortical bone*				
Cortical area %	**0.019 ± 0.002**^**b**^	**0.019 ± 0.002**^**b**^	0.006 ± 0.004^a^	0.002 ± 0.003^a^
Total area %	**0.012 ± 0.002**^**b**^	**0.009 ± 0.001**^**b**^	0.002 ± 0.003^a^	0.004 ± 0.002^a^
Medullary area %	0.002 ± 0.003^b^	**− 0.009 ± 0.003**^**ac**^	− 0.008 ± 0.007^ab^	0.007 ± 0.005^b^

*Cancellous bone*				
BV/TV %	**0.019 ± 0.006**	**0.022 ± 0.006**	0.005 ± 0.013	0.000 ± 0.009
Tb.Th %	**0.014 ± 0.003**^**a**^	**0.023 ± 0.003**^**b**^	0.006 ± 0.007^a^	**0.010 ± 0.004**^**a**^
Tb.N %	0.004 ± 0.004	− 0.002 ± 0.005	0.012 ± 0.010	− 0.011 ± 0.007
Tb.Sp %	0.006 ± 0.004	− 0.001 ± 0.004	0.012 ± 0.007	− 0.006 ± 0.004

**Table 4:** The slope of the strain:response curves representing the responsiveness of each bone parameter to increasing mechanical strains in male mice (mean ± SE, n = 8/group) in response to loading. A significant interaction between the slope of the adaptive strain–response curve and genotype was detected for cortical area and trabecular thickness indicating an effect of genotype on gradients of the slope (p < 0.01 to < 0.001). Different letters in a row denotes the groups are significantly different to each other, as detected from the post-hoc analysis (p < 0.05 to < 0.001). Slopes in bold are significantly different from zero.

	M *WT*^*HBM−*^	M *Lrp5*^*HBM+*^	M *WT*^*+/+*^	M *Lrp5*^*−/−*^
*Cortical bone*				
Cortical area %	**0.024 ± 0.003**^**c**^	**0.019 ± 0.001**^**c**^	**0.015 ± 0.006**^**ac**^	0.001 ± 0.006^ab^
Total area %	**0.011 ± 0.002**	**0.009 ± 0.003**	**0.009 ± 0.004**	0.003 ± 0.004
Medullary area %	0.005 ± 0.003	− 0.007 ± 0.004	− 0.002 ± 0.006	0.006 ± 0.006

*Cancellous bone*				
BV/TV %	**0.023 ± 0.008**	**0.039 ± 0.010**	0.015 ± 0.015	0.008 ± 0.015
Tb.Th %	**0.018 ± 0.005**^**a**^	**0.037 ± 0.006**^**b**^	**0.020 ± 0.009**^**ab**^	0.002 ± 0.009^a^
Tb.N %	0.003 ± 0.004	0.002 ± 0.006	− 0.003 ± 0.008	0.003 ± 0.009
Tb.Sp %	− 0.004 ± 0.003	**− 0.010 ± 0.005**	− 0.005 ± 0.007	− 0.002 ± 0.007
